# Sex-Specific Transcriptome Signatures in Pacific Oyster Hemolymph

**DOI:** 10.3390/genes16091033

**Published:** 2025-08-30

**Authors:** Jingwei Song, Odile V. J. Maurelli, Mark S. Yeats, Neil F. Thompson, Michael A. Banks, Bernarda Calla

**Affiliations:** 1 Department of Fisheries, Wildlife and Conservation Sciences, Coastal Oregon Marine Experimental Station (COMES), Hatfield Marine Science Center, Oregon State University, Newport, OR 97365, USA; 2 Pacific Shellfish Research Unit, United States Department of Agriculture, Agricultural Research Service, Newport, OR 97365, USA

**Keywords:** sex determination, *Magallana gigas*, oyster, hemolymph, gametogenesis

## Abstract

Background/Objectives: Sex determination and differentiation exhibit remarkable molecular diversity across taxa, driven by genetic, epigenetic, and environmental factors. Invertebrates with sequential hermaphroditism, such as the Pacific oyster (*Magallana gigas*), represent a poorly understood system despite their role as keystone species and contribution to a substantial aquaculture industry. Methods: To identify sex-related molecular markers during gametogenesis, we repeatedly sampled hemolymph from artificially conditioned oysters over two months, and sex phenotypes were assigned at the end of the experiment by biopsy. Results: RNA-sequencing analysis of five males and five females revealed subtle yet consistent sex-specific transcriptional signatures in hemolymph. We show that gametogenesis proceeds asynchronously among oysters, even within the same sex individuals. Complex physiological trade-offs were discovered between sexes during gonad maturation; in early stages of sexual maturation, females prioritized cell division, whereas males suppressed it. Females exhibited higher expression of solute carrier family (SLC) genes, suggesting enhanced nutrient exchange during oogenesis. Temporal dynamics highlighted differential expression of genes regulating cross-membrane ion gradients (e.g., transient receptor potential channels) and signal transduction (e.g., signal transducer and activator of transcription), previously linked to environmental sex determination (ESD) in some reptilian species. Conclusions: Together, these findings underscore that gametogenesis in Pacific oysters is complex and dynamic, and that molecular pathways of ESD may be partially conserved between invertebrate and vertebrate species.

## 1. Introduction

Sex determination is a fundamental biological process that guides the development of reproductive organs and is critical for the survival of species dependent on sexual reproduction. The mechanisms of sex determination are broadly categorized into two major types: genetic sex determination (GSD) and environmental sex determination (ESD) [1]. In GSD, sex determination is typically dependent on the presence of a heteromorphic sex chromosome and initiated by differential regulation of one or a few master sex-determining genes. Well-studied examples of sex determining genes include the *SRY* gene in mammals [2] and the *DMRT1* gene in birds [3], which direct gonadal development. In contrast, ESD relies on external environmental cues—such as temperature or social factors—to influence sex outcomes. In many reptiles, egg incubation temperature determines whether an embryo develops as male or female [4], whereas in some fish species, social hierarchy and stress hormones can trigger sex reversal [5,6].

Despite widespread occurrence across diverse taxa, ESD remains poorly understood compared to GSD, primarily due to the complex interplay between environmental variables and developmental plasticity. Identifying causal environmental triggers and how these signals are transduced into molecular and physiological outcomes poses significant challenges. Moreover, while GSD has been extensively studied in many model organisms, research on ESD has been restricted to a handful of vertebrate species, such as reptiles and fishes [7]. This vertebrate-centric focus limits our understanding of the evolutionary origins and diversity of ESD mechanisms. Given that ESD has independently evolved multiple times across different metazoan lineages, expanding research into non-vertebrate species may provide crucial insights into the evolutionary significance of these environmentally influenced systems.

The Pacific oyster, *M. gigas* (formerly *Crassostrea gigas*), is a marine bivalve mollusk that is extensively cultured worldwide except Antarctica. Pacific oysters exhibit irregular sequential hermaphroditism, meaning individuals can randomly switch between male and female sexes across successive reproductive cycles [8]. Despite extensive research, the mechanisms for sex determination in this species remain enigmatic. Pacific oysters lack a sex chromosome, and although genetic effects on sex determination have long been hypothesized [9,10], no definitive sex-determining genes nor consistent genetic markers have been identified to date.

Environmental factors appear to play a significant role in determining sexual phenotypes in Pacific oysters. Variables such as temperature [11,12], food availability [13,14], population density [15], and pH [16] have been shown to influence sex ratios. This environmental plasticity suggests a complex interplay between external factors and intrinsic regulatory pathways. Previous studies have found conserved sex-related genes in Pacific oyster gonads, including orthologs of mammalian sex determination pathway regulators such as *Foxl2* [17,18], *Dmrt1-like* [19,20], and *Sox-like* [21,22]. These findings imply that while the upstream triggers of sex determination may differ, downstream molecular pathways might be evolutionarily conserved with the more well-studied model organisms.

Nevertheless, a comprehensive model explaining how an individual Pacific oyster transitions from a bipotent gonad to producing oocytes or spermatocytes remains elusive. For previous studies, oysters were lethally sampled during various gametogenesis stages [18,23,24]. Repeated gonad sampling has been attempted in the Pacific oyster, but has resulted in highly variable mortality rates (0% to 90%) [22,25]. Gonad sampling could negatively impact gametogenesis. Repeated sampling of the same individuals throughout gametogenesis could better reflect physiological patterns, as well as controlling for individual variability [26]. Hemolymph plays multiple important roles in oyster innate immunity, nutrient transport, and hormone regulation [27,28] and can be nonlethally collected from bivalves. In the Eastern oyster (*C. gigas*), hemolymph protein assays were used to differentiate sex [29]. We hypothesized that the hemolymph transcriptome might display signatures for sex phenotype in the Pacific oyster. The purpose of this study is to determine whether there are sex-specific gene expression signatures in hemolymph over the course of gametogenesis.

## 2. Materials and Methods

### 2.1. Animal Husbandry

Adult *M. gigas* (*n* = 50) were randomly selected from 5-year-old animals (Miyagi population) of the Molluscan Broodstock Program (MBP) at the Oregon State University (OSU) Hatfield Marine Science Center, in Newport, Oregon [30]. All procedures were performed in compliance with university guidelines on animal research.

**Figure 1:**
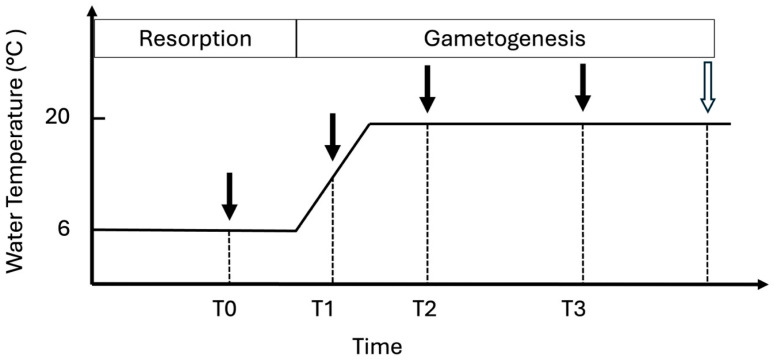
Experimental timeline. Hemolymph from fifty Pacific oysters (*M. gigas*) was sampled at four time points (black arrows) on days 22 (T0), 36 (T1), 51 (T2), and 79 (T3) of an artificial gonad conditioning period. All oysters were phenotypically sexed on day 99 (white arrow). *Y*-axis is water temperature (°C).

At the start of the experiment (day 0), oysters were kept at 6 °C to induce gamete resorption from the previous spawning season. Beginning on day 33, seawater temperature was gradually increased at a rate of 2 °C per day until it reached 20 °C, at which point the temperature was kept constant for the remainder of the experiment (Figure 1). From the time the water temperature reached 20 °C, oysters were fed a mix of *Chaetoceros muelleri* (~60%) and *Tisochrysis lutea* (~40%) continually dosed into a common head tank and manifolds delivered algae-enriched seawater at a rate of 2 L/min and an inflow algal density of ~125,000 cells/mL. The two algal species were commonly used in oyster hatcheries to support optimal growth of animals. Pure cultures of both algae species were grown in F/2 medium (Pentair Aquatic Eco-Systems, Apopka, FL, USA) with a salinity of 34 ppt and held at 21 °C in photobioreactors (Industrial Plankton Photobioreactors Inc, Victoria, BC, Canada) with a light intensity of ~1500 µmol m^−2^ s^−2^ without a photoperiod. Algal concentrations were quantified via counts done using light microscopy.

### 2.2. Sample Collection

Oysters were anesthetized in an 8% (*w*/*v*) magnesium sulfate USP (heptahydrate) bath, prepared by dissolving 4 kg in 25 L deionized water and diluting to 50 L with 1 micron-filtered ambient seawater (salinity 25–35 ppt). Hemolymph was collected four times, on day 22 (“T0”), day 36 (“T1”), day 51 (“T2”), and day 79 (“T3”) (Figure 1), flash-frozen in liquid nitrogen, and stored at −80 °C until RNA extraction. A sterile 1 mL syringe and needle (30 G, 25 mm, BD, Franklin Lakes, NJ, USA) were used to collect hemolymph samples (~100–200 µL) from each oyster.

### 2.3. Sexing

Sexing of oysters occurred 20 days after the final hemolymph sampling, on day 99 (Figure 1). Animals were first anesthetized using the same protocol as above, after which a micro-hematocyte capillary tube (1.1 mm I.D., VWR, Radnor, PA, USA) was used to make a small incision in the gonad to release gametes. The gametes were then transferred to a glass slide, supplemented with a drop of filtered seawater, and observed under a light microscope (DM1000, Leica, Wetzlar, Germany) at 100–400× magnification. Sex for each oyster was recorded as either male, female, hermaphrodite, or N/A (if neither oocytes nor sperm were observed).

### 2.4. RNA Extraction

RNA was extracted using the Monarch Total RNA Miniprep (New England Biolabs, Ipswich, MA, USA) following the mammalian whole blood protocol, which included on-column DNase digestion (final elution volume = 30 µL). RNA concentration and purity were measured using a NanoDrop 2000 (Thermo Fisher Scientific, Waltham, MA, USA), a Qubit 2.0 Fluorometer (Thermo Fisher Scientific, USA) using High Sensitivity RNA reagents, and on an Agilent 2100 Bioanalyzer with RNA 6000 Nano chips (Agilent Technologies, Santa Clara, CA, USA).

### 2.5. RNA-Sequencing Analysis

Hemolymph samples from the same 10 oysters (5 females and 5 males) at each of the four time points were selected based on RNA quantity (RNA concentration ranging from 4.2 to 43.2 ng/ul based on Qubit readings) and visual examination of RNA traces of Bioanalyzer results. Samples were used for paired-end RNA-seq with a minimum targeted 20 million reads per sample. RNA libraries were constructed and sequenced at the University of Illinois Roy J. Carver Biotechnology Center. Libraries were generated with the Kapa Hyper Stranded mRNA library kit (Roche, Basel, Switzerland) and sequenced on a NovaSeq X Plus (Illumina, USA) using a 151-cycle V1.0 kit. Resulting FASTQ files were demultiplexed with the bcl2fastq v2.20 Conversion Software (Illumina, San Diego, CA, USA).

### 2.6. Bioinformatic Analyses

The Pacific oyster genome (‘cgigas_uk_roslin_v1’) and annotation files (v1.59) were downloaded from Ensembl Metazoa (https://metazoa.ensembl.org/Crassostrea_gigas/Info/Index, accessed on 24 May 2024) [31]. Read quality was checked using FastQC V0.11.9 [32], and no trimming was necessary per FastQC output. A genome index was built using HISAT2 v2.2.1 [33]. RNA-seq reads were aligned to the indexed genome with HISAT2 default parameters to generate SAM files for each sample. SAM files were converted to BAM format and sorted using SAMtools v1.6 [34]. Read counting was done using HTseq-count v2.0.2 [35] with settings for stranded libraries. To assess the quality of the overall dataset, the raw count matrix was filtered to remove only the lowest expressed genes (genes with counts per million (CPM) < 0.5 in more than 1 sample were discarded) and normalized by the built-in variance stabilization (VST) function in DESeq2 v1.42 [36]. VST transformation uses the experiment-wide trend of variance over mean, in order to transform the data to remove the experiment-wide trend [37]. Principal component analysis (PCA) was run using the VST count matrix and plotted in DESeq2 to assess sample clustering and outliers. Pearson correlation coefficient among all sample pairs was calculated using the cor() function in R 4.5.0 [38]. A heat map was generated with the R package pheatmap v.1.0.12 [39], using the “average” clustering method.

### 2.7. Pairwise Comparisons Between Sexes

To gain insights into how gene expression differs between sexes at each time point, gene counts were imported into R, and low-count genes were removed using the CPM criteria previously described. Sex was used as a covariate in constructing DESeq2 models using male samples as a reference. Estimation of size factors, dispersion, and differential expression test based on the negative binomial distribution was performed by DESeq2 default for each time point. Differentially expressed genes (DEGs, |log_2_ fold-change| > 1, FDR < 0.05) were used for downstream analyses.

### 2.8. Pairwise Comparisons Between Time Points

To gain insights into how gene expression changed over time within each sex, gene counts were imported into R, and low-count genes were filtered as above. Time and individual oyster were used as covariates in DESeq2 models, and the earlier time point was used as reference (e.g., T1 vs. T0, T0 is the reference; T2 vs. T1, T1 is the reference). Estimation of size factors, dispersion, and differential expression test based on the negative binomial distribution was performed by DESeq2 default for each time point. DEGs (|log_2_ fold-change| > 1, FDR < 0.05) were used for downstream analyses. For both females and males, unsupervised clustering of temporal gene expression was performed using Mfuzz [40] as implemented in MultiRNAflow [41], with membership = 0.5, Min.std = 0.1. All other parameters were kept as defaults.

### 2.9. Biological Pathways and Processes from DEGs (topGO)

Gene ontology (GO) classes for each gene were retrieved using PANNZER [42]. GO enrichment analysis was done using topGO v.2.54.0 [43]. DEGs from each pairwise comparison between sexes and time points (except for T0, due to low numbers of DEGs) above were the “genes of interest” used in GO analyses. All genes with a GO biological process term were the “gene universe” used in GO analyses. Enrichment was tested with Fisher’s exact test (α = 0.05).

### 2.10. Gonad Transcriptome Reanalysis with Data from Public Databases

The authors previously reanalyzed an RNA-seq dataset from Broquard et al. [24], who used female and male Pacific oyster gonads from stage 0 (“undifferentiated”), stage 1 (“gonia proliferation”), and stage 3 (“ripe gonad”) in order to identify sex determining genes. Part of the new results from the reanalysis was included to help put the current hemolymph study into perspective. The oysters used in Broquard et al. (2021) [24] did not change sex in the previous four years and thus were considered “true males” or “true females”. Raw RNA-sequencing reads were downloaded (NCBI accession: PRJNA660750) and remapped to the Peñaloza et al. (2021) [31] Pacific oyster genome assembly; the count matrix was generated, filtered, and clustered with PCA as described above.

## 3. Results

Over the course of the experiment, a total of fifty adult Pacific oysters were repeatedly anesthetized and sampled for hemolymph. Samples were taken at four distinct time points spanning the entire gametogenesis period when maturing in a controlled aquaculture system. Not all oysters were able to be sampled at each time point, either due to a lack of sufficient anesthetization (did not open) or insufficient volume of hemolymph that could be extracted. A total of 20 oysters had samples from all four time points, 10 of which (5 males, 5 females) were selected for analysis based on RNA quality. In total, 38 samples were used in bioinformatic analyses (excluding two T0 female samples, which failed during RNA-seq library preparation).

**Figure 2:**
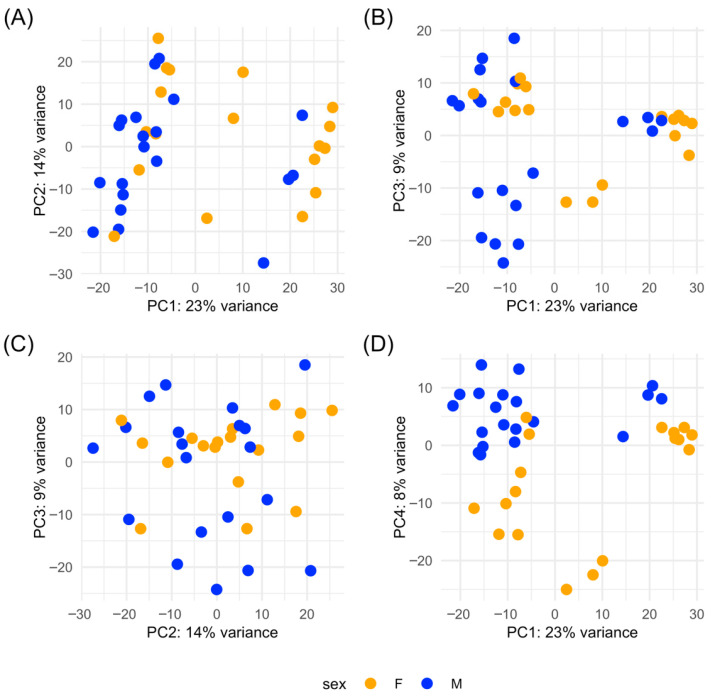
Principal component analysis (PCA) of hemolymph RNA-seq data from adult *M. gigas* (*n* = 38) during artificial gonad maturation: (**A**) PC1 vs. PC2; (**B**) PC1 vs. PC3; (**C**) PC2 vs. PC3; (**D**) PC1 vs. PC4. Orange = females, blue = males.

**Figure 3:**
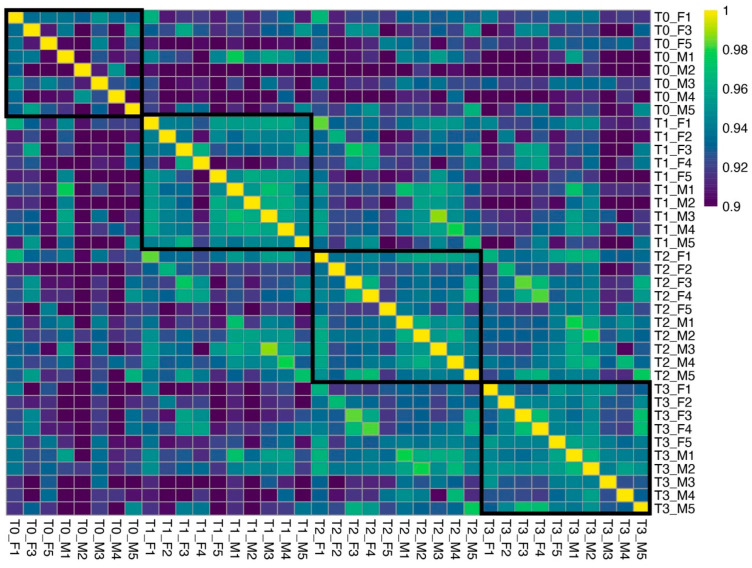
Pearson’s correlation between samples of *M. gigas* (*n* = 38). Samples were ordered based on time points (T0, T1, T2, and T3, black squares). Values lower than 0.9 were cutoff to enhance contrast.

Sequencing resulted in a mean total reads per library of 33.2 million, with a mean mapping rate of 80.7% (Table S1). A principal component analysis showed that the first three principal components explained 46% of the total variance (Figure 2). Female–male separation was the most evident when plotting PC1 with PC4 (Figure 2D). Genes with the highest loadings on PC4 included spermatogenesis-associated protein 7 (G13485), radial spoke head 10 homolog B (G1573), and dynein heavy chain 3-axonemal (G30136), all of which have putative functions in ciliary movement. In addition, two HECT-type E3 ubiquitin transferases (G19927, G33006) and an E2 ubiquitin-conjugating enzyme (G5991) had GO terms related to protein polyubiquitination. Samples did not cluster by time point. A between-sample correlation analysis found that T0 samples showed the lowest correlations among each other compared to samples in T1, T2, and T3 (Figure 3). T1 males (including a female, F5) and T2 males formed discernible clusters, but females did not. Some oysters sampled at adjacent time points tended to have the highest correlations in gene expression, such as M1 at T0 and T1, F1 at T1 and T2, and M3 at T1 and T2 (Figure 3).

### 3.1. Pairwise Comparisons Between Time Points

**Figure 4:**
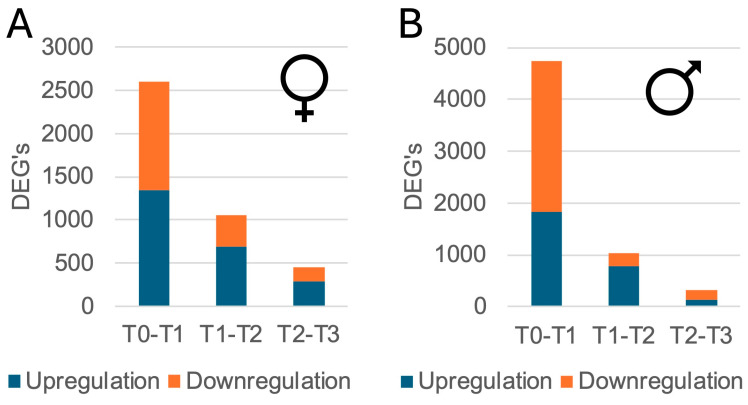
Number of differentially expressed genes (DEGs) in hemolymph of *M. gigas* between adjacent time points for females (**A**) and males (**B**).

When comparing samples of the same sex between adjacent time points, the largest number of DEGs was found between T0 and T1 (F: 2600; M: 4733), followed by T1 to T2 (F: 1058; M: 1024) and T2 to T3 (F: 458; M: 316) (Figure 4). These DEGs are shown on volcano plots, and the top 10 genes with the lowest *p*-values are highlighted (Figure S1). During early time points (T0 to T1), females upregulated genes enriched in cell cycle and DNA replication processes (e.g., GO:0006260, GO:0007049), while ribosome biogenesis-related pathways were downregulated (e.g., GO:0022613, GO:0042254) (Table S2). From T1 to T2 in females, pathways such as stabilization of membrane potential (GO:0030322), G-protein signaling (GO:0007186), and neuropeptide signaling (GO:0007218) were upregulated, while cell cycle and DNA replication pathways were downregulated (GO:0007049, GO:0006260). In later time points (T2 to T3) in females, protein refolding (GO:0042026) and several signaling pathways (GO:2001235, GO:0016055) were upregulated, while epigenetic gene silencing (GO:0031047, GO:0031507) and immune responses were downregulated (GO:0006955) (Table S2).

In early time points (T0 to T1) in males, immune pathways (e.g., GO:0002376, GO:0006955) and protein folding (GO:0006457) were upregulated (Table S2). Genes in positive regulation of cytosolic calcium concentration (GO:0007204) were upregulated, while genes in calcium ion transmembrane transport (GO:0070588) were downregulated. The latter included several genes encoding transient receptor potential (TRP) channels, many of which showed sharper declines in males compared to females (Figure S2). In addition, the Janus kinase/signal transducer and activator of transcription (JAK/STAT) signaling pathway (GO:0007259, GO:0097696) was a top GO term in males, which included signal transducer and transcription activators (STATs) and tyrosine kinases (Figure S3). Meanwhile, genes in cilium organization (GO:0044782) and cell population proliferation (GO:0008283) were downregulated. From T1 to T2, upregulated genes in males were enriched in monoatomic ion transport (GO:0034220) and cell adhesion (GO:0007155), while immune response (GO:0006955) was downregulated. In later time points (T2 to T3) for males, genes in protein localization (GO:0045053, GO:0032507) and sulfation (GO:0051923) were upregulated. Similar to females, epigenetic gene silencing (GO:0031047, GO:0031507) and immune response (GO:0006955) were downregulated (Table S2).

### 3.2. Temporal Clustering Analysis

Unsupervised clustering identified four optimal temporal clusters for females and three optimal clusters for males (Figure S4). T1 was clearly identified as an inflection point in all clusters. Gene IDs belonging to each cluster can be found in Table S3.

### 3.3. Pairwise Comparisons Between Sexes

**Figure 5:**
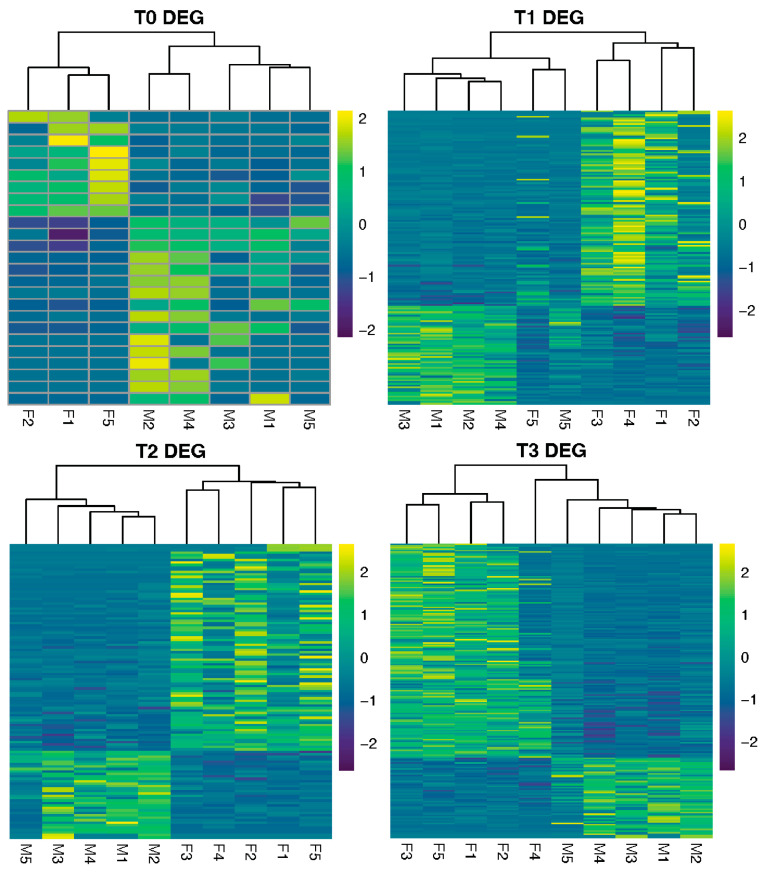
Row-scaled gene expression values (Z-score) for differentially expressed genes (DEGs) (FDR < 0.05, |log2FC| > 1) between sexes, separated by time point sampled (T0, T1, T2, and T3).

Far fewer DEGs were found between sexes than between time points, with a total of 501 nonredundant DEGs detected between sexes across all time points. T0 had the lowest and T3 had the highest number of DEGs (25 and 191, respectively). Besides T0, there were more DEGs with higher expression in females than in males at each time point. These DEGs are shown on volcano plots, and the top 10 genes with the lowest *p*-values are highlighted (Figure S5). Two genes were found to be differentially expressed between sexes across all time points: a gene encoding for a “Toll-like receptor 2 type-2” (G30751) and another encoding for “prestin” (G30219), both of which were upregulated in males relative to females except for M5 (Figure S6). If T0 samples were not included, there were only 20 annotated DEGs shared among T1, T2, and T3 between sexes (Table S4). When samples were clustered based on Z-scores of these DEGs, M5 stood out as an outlier compared to other males (Figure 5).

Gene ontology enrichment analysis showed that in T1, T2, and T3, genes upregulated in females were significantly enriched for various “transport” terms (e.g., GO:0006810, GO:0055085, GO:0015711), including many solute carrier family (SLC) proteins (Figure S7). The “transmembrane transport” at T3 was the most statistically significant among all time points (*p* = 8.80 × 10^−11^). Chitin metabolic processes (e.g., GO:0006032) were significantly upregulated in females at T1 and T2. In males, genes that were upregulated at T1 were enriched in innate immune processes (GO:0045087, GO:0098542). At T2, females upregulated fatty acids and lipid metabolic processes (e.g., GO:0001676, GO:0006629), while the most significant GO terms in males were protein processing and maturation (GO:0016485, GO:0051604). At T3, monoatomic ion transmembrane transport (GO:0034220) was upregulated, including genes such as a “leucine-rich repeat-containing protein 45” (G1897) and a “zinc transporter ZIP4” (G5048) (Table S5).

### 3.4. Gonad Transcriptome Reanalysis

**Figure 6:**
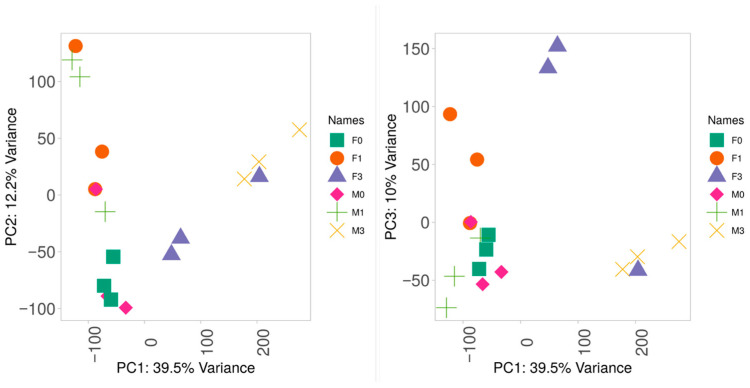
Principal component analysis (PCA) of gonad RNA-seq data from adult *M. gigas* (Broquard et al. 2021) [24], after mapping to a more recent and complete reference genome (Ensembl Metazoa: ‘cgigas_uk_roslin_v1’). F = female, M = male, 0 = immature, 1 = maturing, 3 = mature. Note that the samples’ designations are distinct from hemolymph samples used in the rest of the paper.

By remapping and analyzing a publicly available gonad transcriptome dataset from the Pacific oyster [24], we found that immature gonads (stage 0 and 1) were separated from mature gonads (stage 3) along PC1. In addition, a sample deemed as stage 3 female in this dataset clustered with stage 3 males on the PCA plot (Figure 6).

## 4. Discussion

The main objective of this study was to determine whether there is sex-biased gene expression in hemolymph of Pacific oysters during gametogenesis and explore their potential patterns and functions. Repeated hemolymph sampling was conducted during the entire period of oyster gonad maturation to screen for potential sex- or phase-specific gene expression. We chose to sample hemolymph because of its various roles in oyster physiology and since it could be sampled multiple times from the same individual without overstressing the animal. Only one mortality was observed out of 50 oysters, and normal, mature gametes were observed in all surviving animals at the end of the study when gonads were ripe and ready for spawning.

In previous studies that dealt with sex determination and gonad maturation, oysters were lethally sampled at various gametogenesis stages, sometimes before the phenotypic sex could be determined [18,23,24]. A recent study attempted to circumvent this problem by only including oysters whose sexes remained unchanged in four years, which they referred to as “true males” and “true females” [24], but their results did not separate stage 3 males and females on PCA space (see Figure 1 of Broquard et al. 2021) [24]. Additionally, from our analysis, there is a sex-mislabeled sample that could be either due to a potential sample recording error, or the “true” female could have developed as a male during the study. The same group of authors also reported that all Pacific oysters can develop as either sex and switch between sexes across years [8].

Overall, our results are consistent with the prediction that there is a weak but detectable signal of sex phenotypes in oyster hemolymph, supporting previous research that evaluated sex-specific proteomic signatures in the hemolymph of Eastern oyster, *C. gigas* [29]. Genes with the highest loadings on PC4 (e.g., G13485 and G1573), which distinctly separate females and males, have putative functions in ciliary movement [44]. Functional manipulations of these homologs in other species have impaired sexual reproduction in planthoppers [45] and mice [46].

The cold acclimation period mimics overwintering conditions, and the transcriptome at T0 showed little differentiation between males and females. This agrees with the lack of histological differences between sexes for resting/pre-conditioned oysters [47,48]. A somewhat unexpected finding was that the transcript encoding for paramyosin (G24796), a key oyster adductor muscle component [49], showed higher abundance in females than males at T0. Female oysters are known to grow faster than males [13,50,51]; thus, this could suggest females are more likely to increase body mass during the resting phase than males [24]. Notably, there were more upregulated genes in females compared to males in warm-water time points (T1, T2, and T3), consistent with previous transcriptomic studies comparing Pacific oyster male and female gonads [18]. The higher number of upregulated genes in females may reflect the greater physiological demand of oogenesis compared to spermatogenesis in oysters.

The oyster gonad is a diffuse, non-permanent organ bathed in hemolymph [52]. Hemocytes are hemolymph cells that play important roles in immunity, intracellular digestion, and nutrient transport [27,28]. Oysters have an open circulatory system where hemocytes migrate deep into various tissues to deliver important nutrient molecules. In females, significant GO terms related to “transmembrane transport” could be related to enhanced nutrient exchange capacity compared to males during gametogenesis, which is expected given that oocytes are much larger and nutrient-dense than sperm. Solute carrier family proteins (SLCs) are membrane proteins that transport a wide range of molecules, including sugars, amino acids, and vitamins, to regulate nutrient levels and remove waste [53,54]. The Pacific oyster genome encodes over 500 SLCs [55]. Maturing female oysters likely meet the high energetic demands of oogenesis by upregulating a wide range of membrane transporter genes in hemocytes, thus enabling enhanced capacity for nutrient exchange with tissues and the developing oocytes. Therefore, enhanced nutrient delivery capabilities may be a signature of maturing and near-mature female oysters.

In natural environments, oyster gametogenesis can proceed asynchronously, and spawning can take place multiple times within a reproductive season [56,57]. In our aquaculture conditioning system, with stable temperature and food availability, asynchronous maturation of gonads is still common even within the same cohort (personal observation). This is also reflected in the transcriptomic data, as samples from the same sex had poor correlation within time points, especially the females. Sample M5, for example, was an outlier whose gene expression often deviated from other male oysters (Figure 5, Figure S6). Five months after the initial sexing, M5 was resexed as a hermaphrodite. Thus, hermaphroditism and asynchronous maturation—driven by individual variability—may explain the consistently low numbers of sex-specific DEGs across time points.

Gene homologs found in the gonads of species with environmental sex determination (ESD) were discovered in oyster hemolymph. Transient receptor potential channels (TRPs) are membrane ion channels that open or close in response to temperature [58,59]. TRPs have been shown to play a role in ESD in alligators and lizards [60,61,62,63]. While most of the *TRPs* examined here decreased expression in both sexes in response to the temperature increase from T0 to T1, *TRPM* (G6654) increased in expression in both sexes. From T0 to T1, *TRPV6* (G24388) expression decreased in males, while it increased in females (Figure S2). TRPV6 is special among other TRPs for its calcium selectivity [64]. This could suggest a conserved temperature-dependent sex determination mechanism across invertebrates and vertebrates, as different TRPs are known to exhibit heterogeneous temperature sensitivities [59]. In another study, differential expression of *TRPs* was found during sexual development in the red-eared slider turtle, *Trachemys scripta elegans* [65]. TRPs are likely involved in sex determination pathways through calcium signaling and protein phosphorylation [62,66]. Accordingly, calcium-related gene ontologies were discovered multiple times in this study. In *T. scripta*, it was shown that calcium influx at female-inducing temperature (31 °C) promotes the phosphorylation of signal transducer and activator of transcription 3 (STAT3), which activates the female pathway while suppressing the male pathway [66,67]. In this study, several STAT transcription factors were significantly upregulated early on in both sexes, with males showing a sharper expression increase than females (Figure S3). Thus, TRPs, STATs, and calcium signaling together likely are part of an ancient sex determination pathway, but their mechanisms require further functional investigations in invertebrates such as oysters.

Another group of proteins that were hypothesized to play a role in ESD are heat shock proteins (HSPs). HSPs are molecular chaperones and regulators of transcription factors [68]. Some families of HSPs are present at ambient temperature, but others are strongly upregulated by thermal stress and other environmental stimuli. In the Chinese alligator, *Alligator sinensis,* seven of the 72 *HSPs* showed a sex-biased expression during a temperature-sensitive period of egg incubation [61], suggesting that at least some HSPs play an important role in sex determination. Evidence is accumulating for HSPs’ involvement in sex differentiation and sex change in various taxa such as alligators [69], fishes [6,70,71], and nematodes [72]. In the present study, many HSPs were among the differentially expressed genes discovered between nearby time points. The *HSPs* examined here showed heterogeneous expression, with high expression of G5808 and G5810 as potential markers for sex prediction (Figure S8).

Gene ontology enrichment analyses on DEGs between time points showed complex physiological trade-offs in early gametogenesis, with distinct strategies between sexes. Females prioritized DNA replication and cell division over ribosome biogenesis, whereas males upregulated immune responses while suppressing cell proliferation. Given the high energy demands of these processes [73], such trade-offs are likely unavoidable [74,75]. We hypothesize that female oysters enter early gametogenesis with a greater energy reserve (e.g., glycogen) after overwintering, as their development requires more energy than males [76,77]. The observed increase in cell division in females may facilitate hemocyte expansion, oogenesis, and vitellogenesis [78,79]. Together, these findings suggest different reproductive strategies between male and female oysters during early gametogenesis. This could explain why older oysters are strongly biased towards females [8,9], as their greater baseline energy reserve may favor female development and survival [80].

## 5. Conclusions

Sequential hermaphroditism in oysters is hypothesized to be determined by a mixture of genetic and environmental factors. Using non-lethal, repeated sampling during gametogenesis, we successfully tracked individual oysters to gonad maturity, enabling retrospective assignment of sex phenotype. Pacific oyster hemolymph displayed differentially regulated genes (e.g., *TRPs*, *HSPs*, and *STATs*) between sexes previously associated with environmental sex determination (ESD) in gonads of other species, suggesting potential conserved pathways for integrating external stimuli. These signals may then be relayed intracellularly, where diverse mechanisms could modulate gene expression, biasing gonad development toward producing oocytes or spermatocytes. Hemolymph extraction from large numbers of oysters is labor-intensive and requires extensive practice to obtain sufficient volume, limiting our ability to test a much higher number of oysters that would reduce variability and increase robustness. Nonetheless, we were able to identify candidate diagnostic markers for sex prediction in oyster hemolymph, providing a foundation for future research in additional samples.

## Data Availability

Raw RNA-seq sequencing reads can be downloaded from the National Center for Biotechnology Information (https://www.ncbi.nlm.nih.gov/) under BioProject PRJNA1262499. All R scripts used in data analyses and visualization can be found at: https://github.com/sjwu571/Hemolymph_RNAseq.

## Supplementary Materials

The following supporting information can be downloaded at: https://www.mdpi.com/article/10.3390/genes16091033/s1, Table S1: Summary statistics of 38 hemolymph samples used in this study. Ten individual oysters (5 males, 5 females) were sampled four times during the period of gametogenesis. Two females failed library prep from T0 were not included; Table S2: Outputs from Gene Ontology enrichment analysis between samples of the same sex, between two adjacent time points; Table S3: Gene IDs in each of the clusters in Figure S4; Table S4: Differentially expressed genes (DEGs) between sexes that were shared among T1, T2 and T3; Table S5: Outputs from Gene Ontology enrichment analysis between sexes, within a single time point; Figure S1: Volcano plots highlighting the top 10 most differentially expressed genes (with labels) between females and males at T0 (a), T1 (b), T2 (c), and T3 (d). Positive log2(fold change) indicates higher expression in males; Figure S2: Normalized counts adjusted by size factor by DESeq2 for eight transient receptor potential (TRP) channel genes, for all 38 samples over four time points. *Y*-axis is in a log scale; Figure S3: Normalized counts adjusted by size factor by DESeq2 for six selected genes in the JAK/STAT pathway, for all 38 samples over four time points. *Y*-axis is in log scale; Figure S4: Temporal clustering of genes for females (a) and males (b); Figure S5: Volcano plots highlighting the top 10 most differentially expressed genes between time points within the same sex. Top panels: females, bottom panels: males. T1 vs. T0 (a,d), T2 vs. T1 (b,e), T3 vs. T2 (c,f). Positive log2 (fold change) indicates higher expression in the first time point of the comparisons; Figure S6: Normalized counts adjusted by size factor by DESeq2 for two significantly differentially expressed genes, Toll-like receptor 2 type-2 and prestin, between sexes across all four time points. *Y*-axis is in log scale; Figure S7: Normalized counts adjusted by size factor by DESeq2 for six selected transporter genes, including solute carrier family (SLC) genes, for all 38 samples over four time points. *Y*-axis is in log scale; Figure S8: Normalized counts adjusted by size factor by DESeq2 for heat shock protein (HSP) genes, for all 38 samples over four time points. *Y*-axis is in log scale.
