# Supplementary material for: Sex-Specific Transcriptome Signatures in Pacific Oyster Hemolymph
Source: Genes (Basel). 2025 Aug 30;16(9):1033. doi: 10.3390/genes16091033 (PMC12469842; doi:10.3390/genes16091033)
Supplement: Supplementary file 1 [file genes-16-01033-s001.zip › FigS3.pdf]

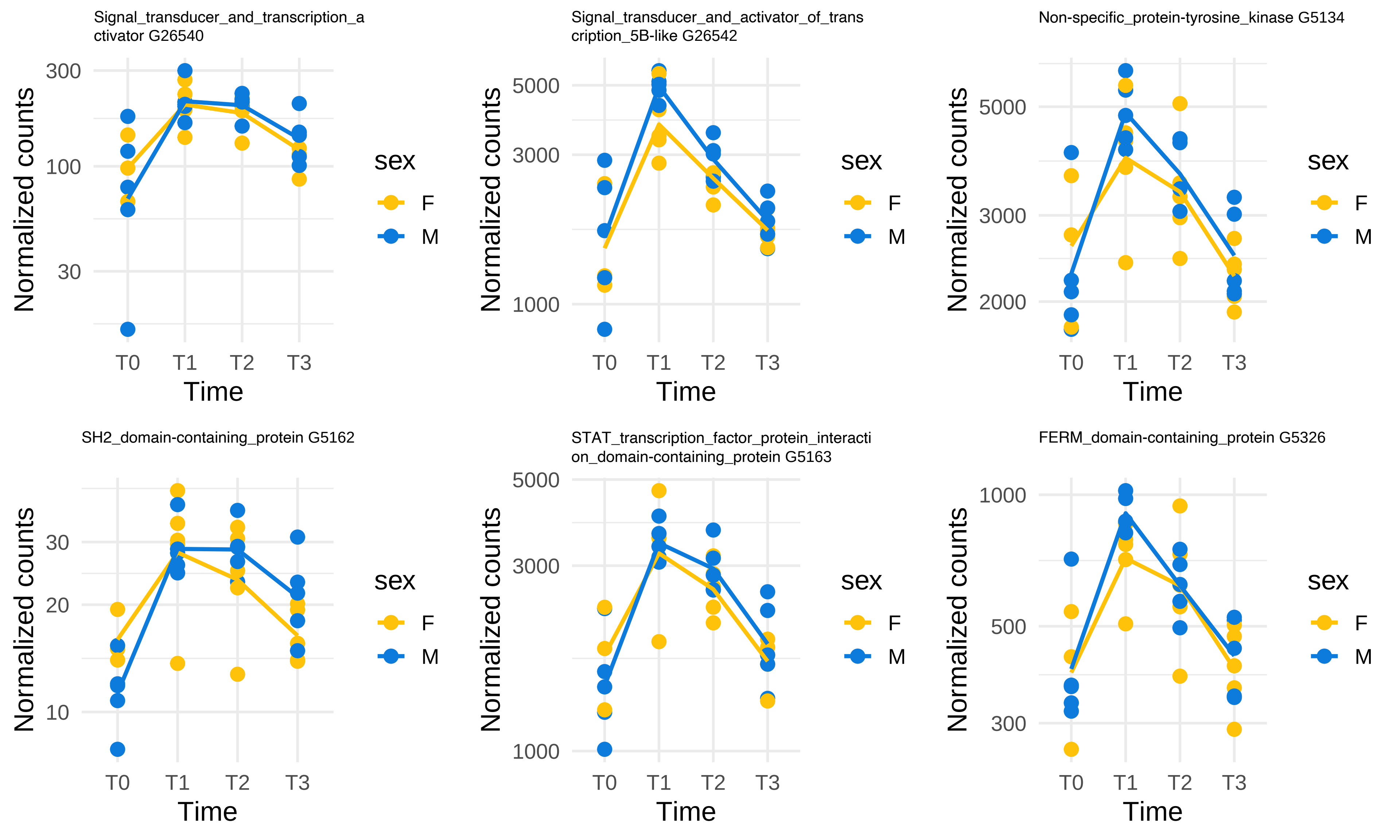

Signal_transducer_and_transcription_a
ctivator G26540
Normalized counts
300
100
30
T0
T1
T2
T3
Time
sex
F
M
Signal_transducer_and_activator_of_trans
cription_5B-like G26542
5000
3000
1000
Non-specific_protein-tyrosine_kinase G5134
5000
3000
2000
SH2_domain-containing_protein G5162
30
20
10
STAT_transcription_factor_protein_interacti
on_domain-containing_protein G5163
FERM_domain-containing_protein G5326
1000
500
300
