# Supplementary material for: Sex-Specific Transcriptome Signatures in Pacific Oyster Hemolymph
Source: Genes (Basel). 2025 Aug 30;16(9):1033. doi: 10.3390/genes16091033 (PMC12469842; doi:10.3390/genes16091033)
Supplement: Supplementary file 1 [file genes-16-01033-s001.zip › FigS7.pdf]

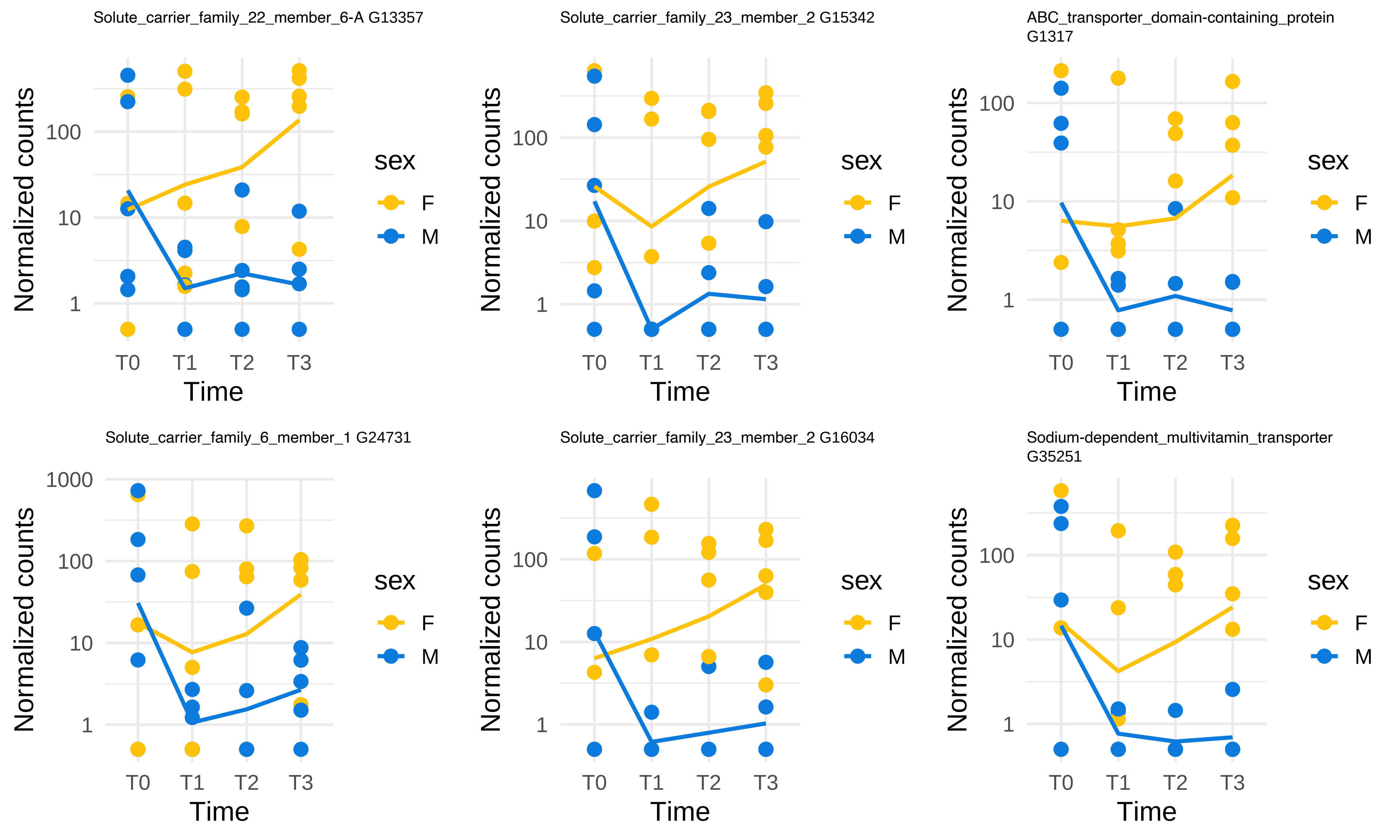

Solute_carrier_family_22_member_6-A G13357
Solute_carrier_family_23_member_2 G15342
ABC_transporter_domain-containing_protein G1317
Solute_carrier_family_6_member_1 G24731
Solute_carrier_family_23_member_2 G16034
Sodium-dependent_multivitamin_transporter G35251
Normalized counts
1
10
100
1000
Time
T0
T1
T2
T3
sex
F
M
