# Supplementary material for: Sex-Specific Transcriptome Signatures in Pacific Oyster Hemolymph
Source: Genes (Basel). 2025 Aug 30;16(9):1033. doi: 10.3390/genes16091033 (PMC12469842; doi:10.3390/genes16091033)
Supplement: Supplementary file 1 [file genes-16-01033-s001.zip › FigS8.pdf]

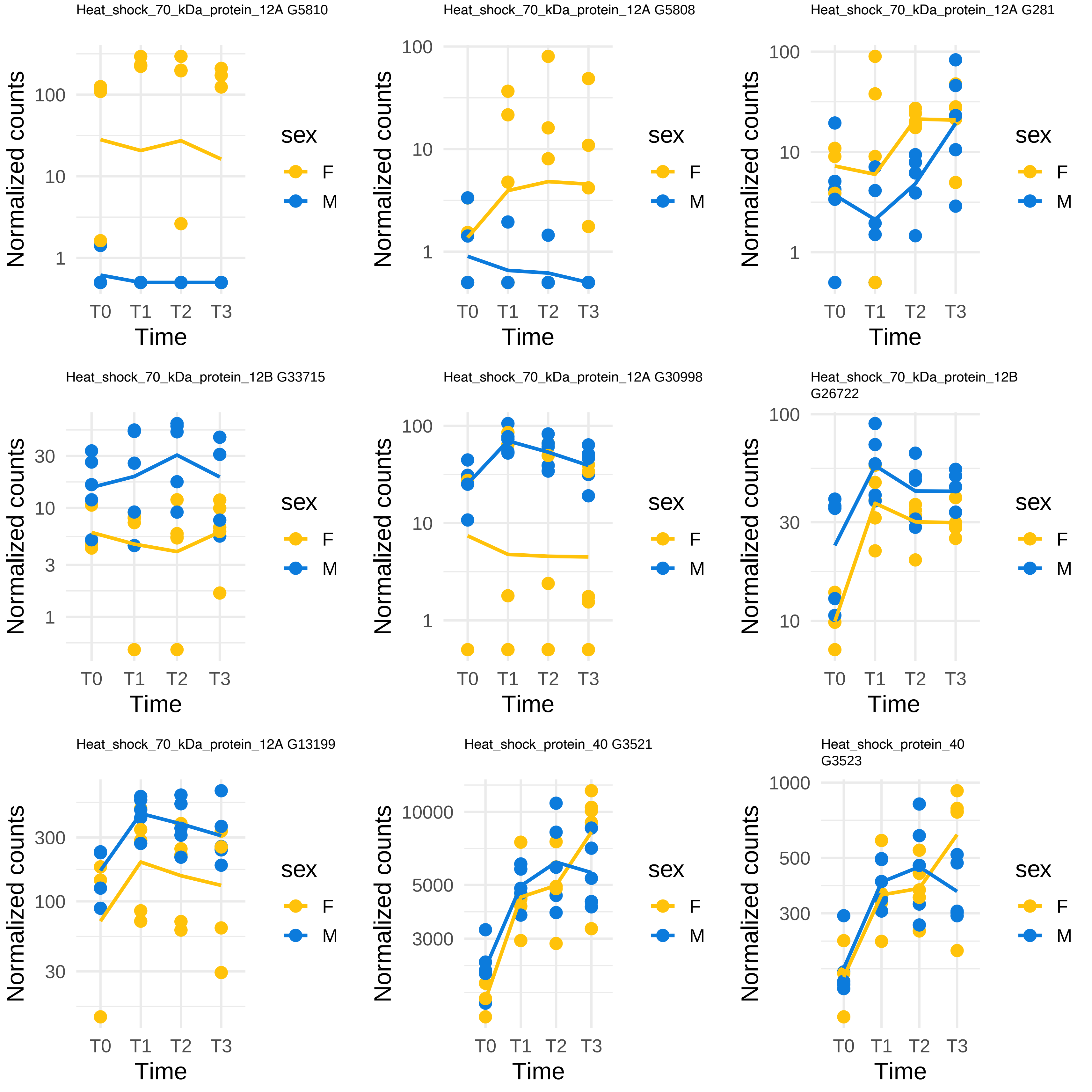

Heat_shock_70_kDa_protein_12A G5810
Heat_shock_70_kDa_protein_12A G5808
Heat_shock_70_kDa_protein_12A G281
Heat_shock_70_kDa_protein_12B G33715
Heat_shock_70_kDa_protein_12A G30998
Heat_shock_70_kDa_protein_12B G26722
Heat_shock_70_kDa_protein_12A G13199
Heat_shock_protein_40 G3521
Heat_shock_protein_40 G3523
Normalized counts
Time
T0
T1
T2
T3
sex
F
M
