# Supplementary figures and images for: Sex-Specific Transcriptome Signatures in Pacific Oyster Hemolymph
Source: Genes (Basel). 2025 Aug 30;16(9):1033. doi: 10.3390/genes16091033 (PMC12469842; doi:10.3390/genes16091033)

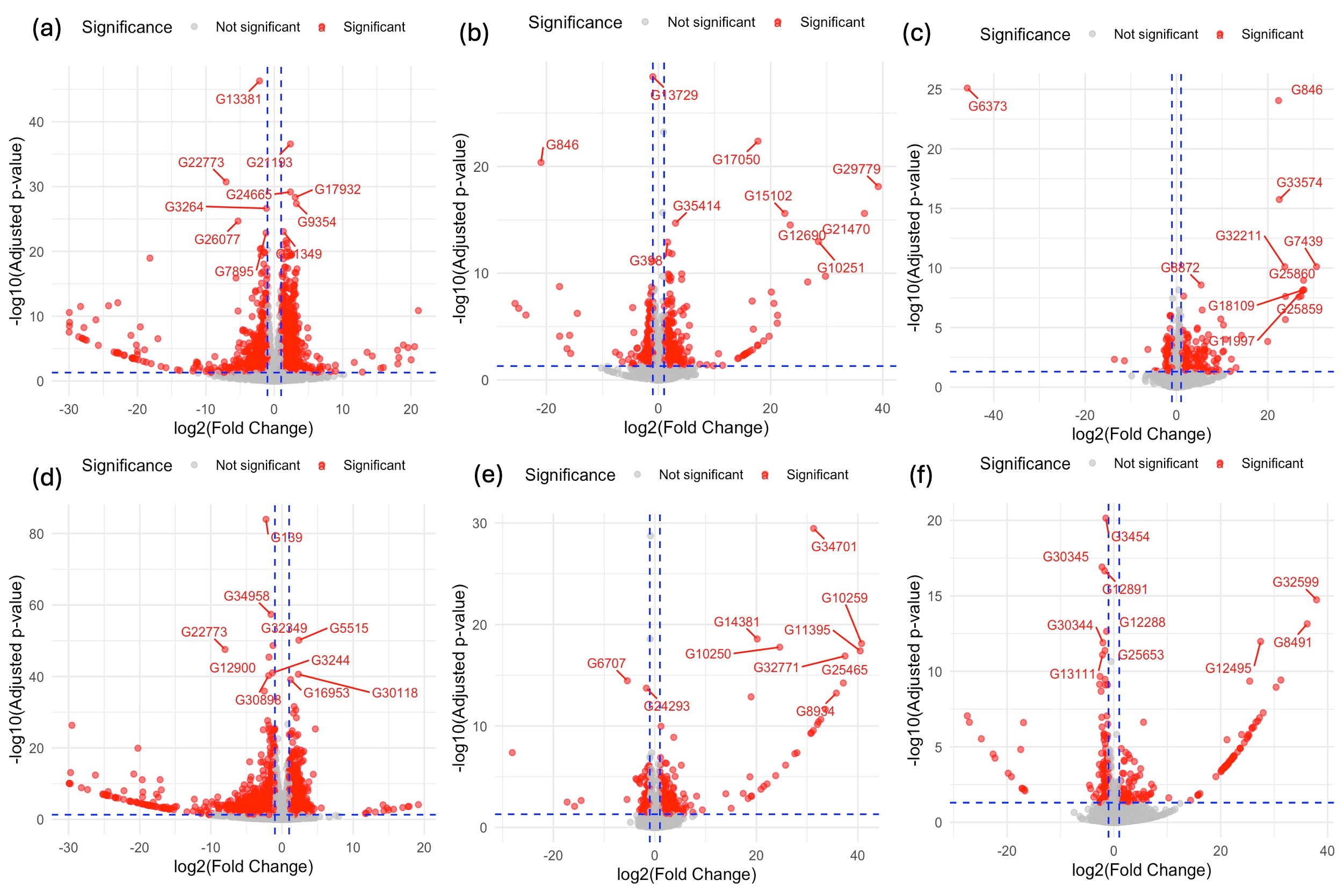

Supplement: Supplementary file 1 [file genes-16-01033-s001.zip › FigS1.jpg]

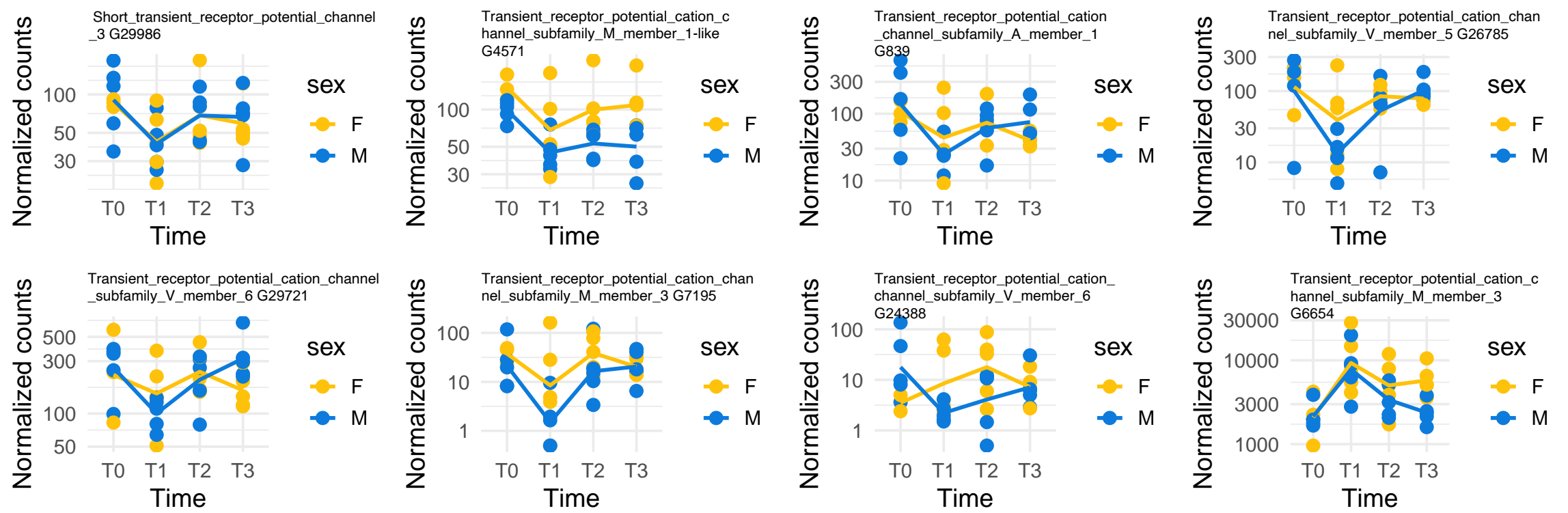

Supplement: Supplementary file 1 [file genes-16-01033-s001.zip › FigS2.pdf]

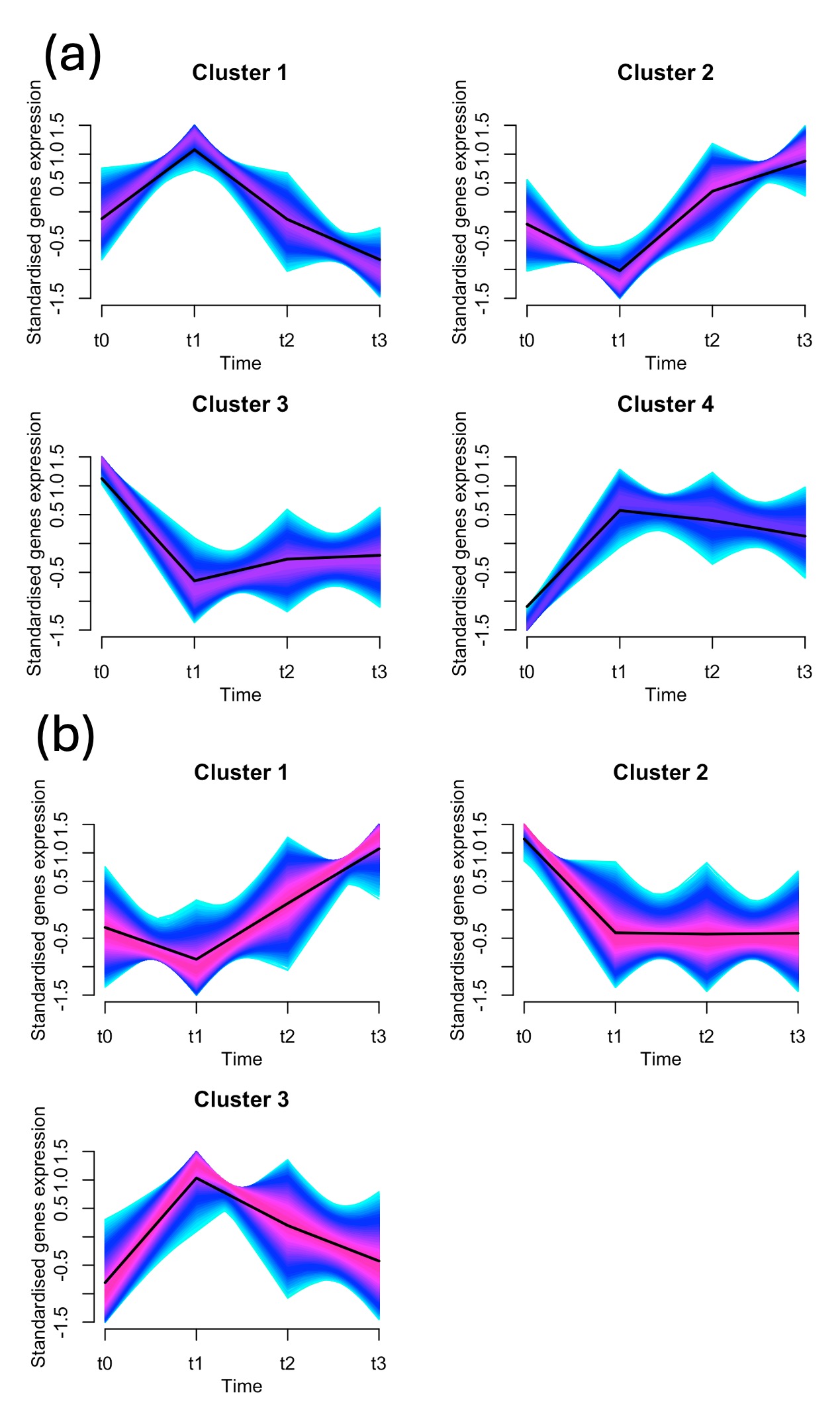

Supplement: Supplementary file 1 [file genes-16-01033-s001.zip › FigS4.jpg]

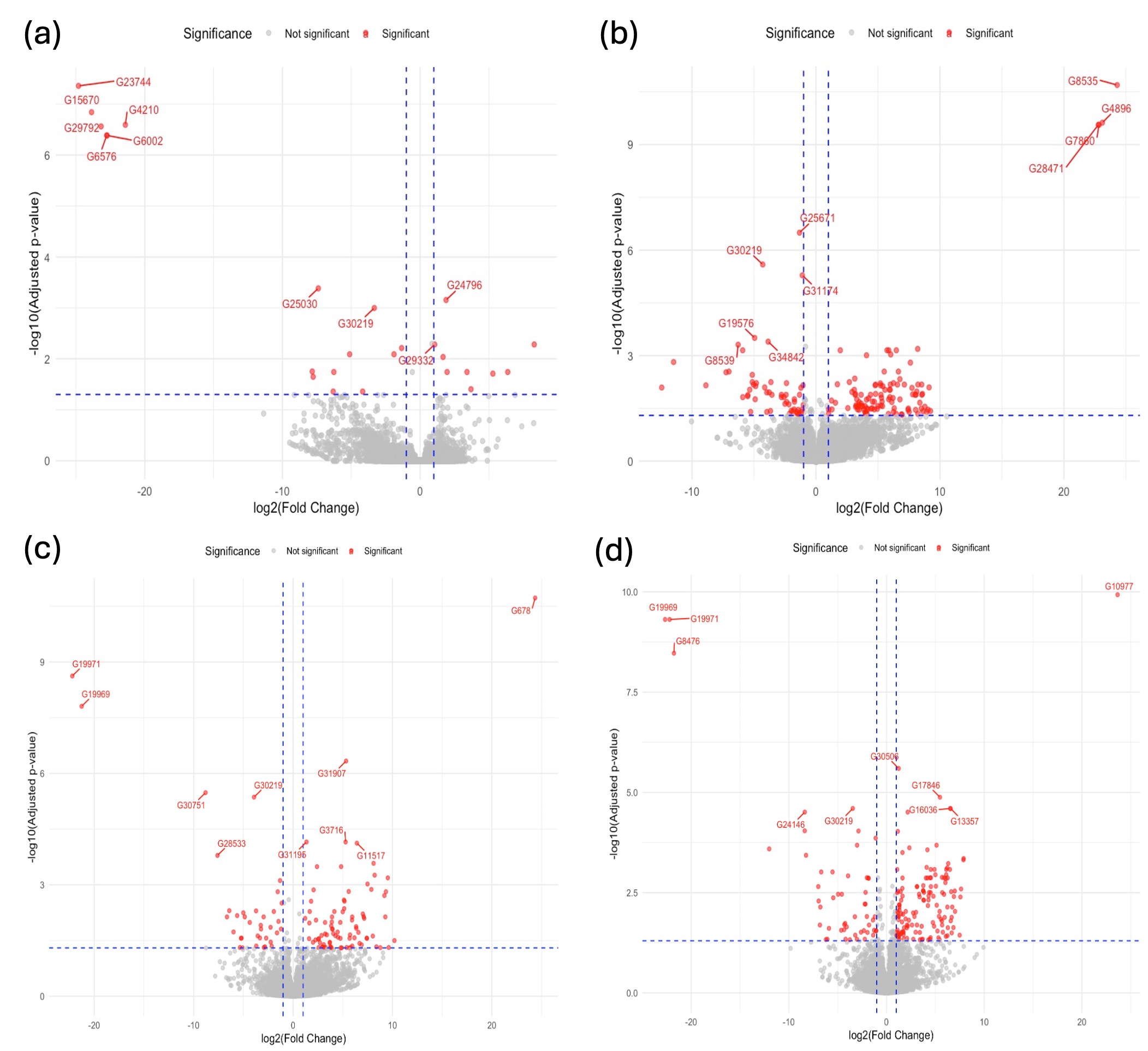

Supplement: Supplementary file 1 [file genes-16-01033-s001.zip › FigS5.jpg]

Toll-like\_receptor\_2\_type-2 G30751

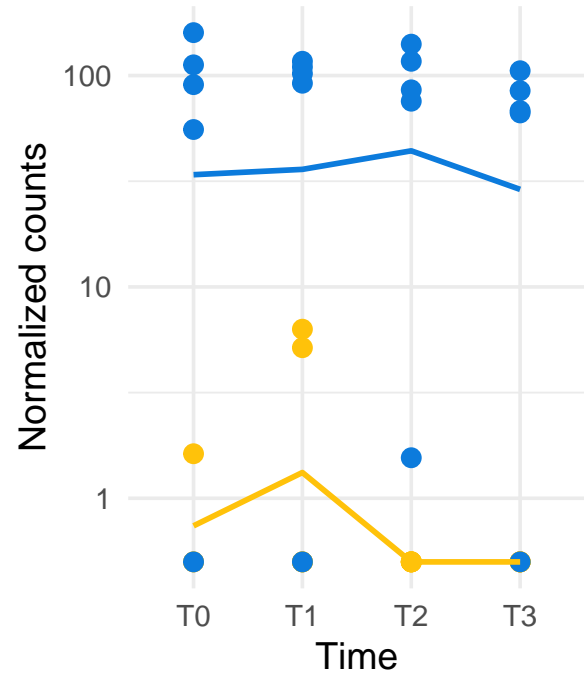

Prestin G30219

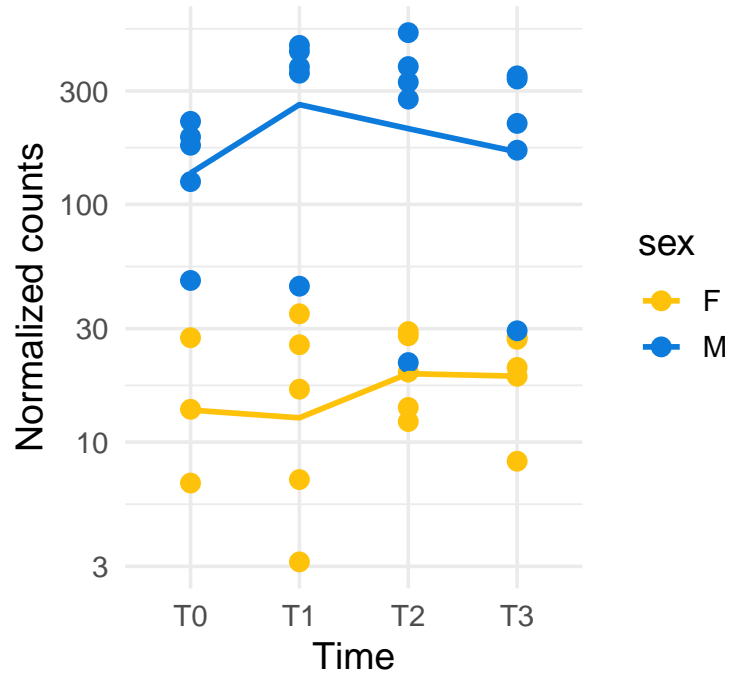

Supplement: Supplementary file 1 [file genes-16-01033-s001.zip › FigS6.pdf]
